# Synthesis of Amorphous MnFe@SBA Composites for Efficient Adsorptive Removal of Pb(Ⅱ) and Sb(V) from Aqueous Solution

**DOI:** 10.3390/molecules30030679

**Published:** 2025-02-04

**Authors:** Zhou Shi, Aogui Zhu, Fan Chen, Yishu Cai, Lin Deng

**Affiliations:** Hunan Engineering Research Center of Water Security Technology and Application, College of Civil Engineering, Hunan University, Changsha 410082, China; shiz61@hnu.edu.cn (Z.S.); zhuaogui1@hnu.edu.cn (A.Z.); chenfan@cmie.cn (F.C.)

**Keywords:** SBA-15, MnFe_2_O_4_, Pb(II), Sb(V), adsorption mechanisms

## Abstract

The extensive release of water contaminated with lead (Pb(II)) and antimony (Sb(V)) constitutes a serious threat to the human living environment and public health, necessitating immediate attention. In this study, a novel MnFe@SBA composite was synthesized using the hydrothermal method through the in situ growth of MnFe_2_O_4_ on SBA-15. The MnFe@SBA exhibits an amorphous structure with a high specific surface area of 405.9 m^2^/g and pore sizes ranging from 2 to 10 nm. Adsorption experiments demonstrated that MnFe@SBA removed over 99% of Pb(II) and 80% of Sb(V) within 120 min at initial concentrations of 10 mg/L, whereas both MnFe_2_O_4_ and SBA-15 exhibited poor adsorption capacities. Additionally, the MnFe@SBA displayed excellent tolerance towards coexisting cations, including Na^+^, K^+^, Mg^2+^, Ca^2+^, Zn^2+^, Ni^2+^, and Cd^2+^, as well as anions such as Cl^−^, NO_3_^−^, CO_3_^2−^, and PO_4_^3−^. The adsorption behavior of Pb(II) onto MnFe@SBA was satisfactorily described by the pseudo-second-order kinetic model and the Freundlich isotherm, while the adsorption of Sb(V) was well-fitted by the pseudo-second-order kinetic model and the Langmuir isotherm. At 318 K, the maximum adsorption capacities of MnFe@SBA for Pb(II) and Sb(V) were determined to be 329.86 mg/g and 260.40 mg/g, respectively. Mechanistic studies indicated that the adsorption of Pb(II) and Sb(V) onto MnFe@SBA involved two primary steps: electrostatic attraction and complexation. In conclusion, the MnFe@SBA is anticipated to serve as an ideal candidate for efficient removal of Pb(II) and Sb(V) from contaminated water.

## 1. Introduction

Lead (Pb(Ⅱ)) is a stable heavy metal existing in the environment and is highly toxic to the human body even at very low concentrations [1,2]. Thus, the World Health Organization (WHO) sets a guideline limit of 10 μg/L for lead in drinking water [3]. Lead can affect the hematopoietic system by inhibiting the synthesis of hemoglobin [4], leading to anemia, and lead poisoning can have serious adverse effects on the nervous system, liver, kidney, and reproductive system [5]. Over the past half-century, more than 800,000 tons of lead have been released worldwide from the battery manufacturing, glass manufacturing, and cosmetics industries [6]. Antimony is another toxic nonessential element, existing primarily in the forms of Sb(III) and Sb(V). Its destructive effects on cellular enzyme activity have serious implications for human health [7]. As its use in the industrial production of flame retardants, batteries, catalysts, and other applications has increased, the concentration of antimony in water has escalated rapidly [8]. The discharge of antimony not only contaminates surface water but also affects sediments, groundwater aquifers, and soil [9]. Furthermore, Sb(III) is readily oxidized to Sb(V) under natural environmental conditions. These environmental and health threats make developing effective methods for removing Pb(II) and Sb(V) from water is an urgent priority.

Commonly used techniques for removing heavy metal ions from aqueous phases include chemical precipitation [10], electrochemical recovery [11], ion exchange [12], membrane separation [13], and adsorption [14]. Adsorption is extensively applied due to its simplicity, energy efficiency, environmental friendliness, and reusability [15]. Graphene oxide [16], chitosan [17], metal–organic frameworks [18], carbon nanotubes [19], and other adsorbents have been studied to remove heavy metal ions from water and wastewater.

SBA-15 is a mesoporous silicon-based material with controllable pore size and high specific surface area, making it suitable to be used as a carrier for adsorbents and catalysts [20,21]. The presence of hydroxyl groups on the inner surface and channels facilitates the adsorption of heavy metal ions by functional nanoparticles immobilized on SBA-15 [22]. Fereshte et al. successfully loaded Fe_3_O_4_ onto mesoporous SBA-15 to prevent nanoparticle agglomeration, resulting in an adsorbent with abundant guanidine and −OH groups, exhibiting maximum adsorption capacities of 344.82 mg/g for Pb(II) and 303.03 mg/g for Cu(II) [23]. Xing et al. incorporated bimetallic Fe/Ni into the six-hole structure of SBA-15 to achieve a remarkable removal efficiency of 97.62% for Cr(Ⅵ) [24]. He et al. uniformly dispersed Cu^0^ onto SBA-15, and the obtained Cu^0^-SBA-15 composite displayed impressive adsorption capacities toward gaseous iodine (954 mg/g) and liquid iodine (842 mg/g) [25].

The recyclability of magnetic materials from aqueous solutions using external magnets makes them highly promising for environmental remediation [26]. Cobalt ferrite (CoFe_2_O_4_) and manganese ferrite (MnFe_2_O_4_) are two typical magnetic nanomaterials commonly applied in catalysis due to their exceptional stability, moderate magnetic saturation, and convenient separation properties [27]. Nevertheless, due to their magnetic properties, nanoparticles of CoFe_2_O_4_ and MnFe_2_O_4_ tend to agglomerate during synthesis, leading to reduced specific surface areas and inadequate adsorption capacities [28]. The incorporation of magnetic nanoparticles into the pore structures of a mesoporous matrix can effectively mitigate agglomeration, thereby enhancing the adsorption capacity toward pollutants. Ren et al. synthesized porous spinel MnFe_2_O_4_ via sol–gel method, and the resulting adsorbent effectively removed Pb(II) and Cu(II) by forming complexes with carboxyl and hydroxyl groups on the surface of MnFe_2_O_4_ [29]. Verma et al. loaded MnFe_2_O_4_ onto graphene oxide through the hydrothermal method, and the as-synthesized composite achieved a remarkable removal efficiency of 99.99% for Pb(II). In the adsorption process, hydroxyl and carboxyl groups are the main functions [30]. Yang et al. constructed a Fe–Mn bimetallic composite, which demonstrated an impressive adsorption capacity of 95 mg/g for Sb(V), and the main mechanism of adsorption is the inner sphere complexation between Sb(OH)_6_^−^ and the exposed hydroxyl groups [31]. Although significant progress has been made in utilizing ferrite materials as adsorbents for the removal of heavy metal ions, limited research has been conducted on the construction of MnFe_2_O_4_ loaded SBA composite for adsorptive removal Pb(II) and Sb(V).

In this study, a MnFe_2_O_4_-loaded SBA composite (MnFe@SBA) with a high specific surface area and excellent adsorption capacity was prepared for the first time using a hydrothermal method. The morphology, crystal structure, and physicochemical properties of the MnFe@SBA were characterized using scanning electron microscopy (SEM), X-ray powder diffraction (XRD), Brunauer–Emmett–Teller (BET) analysis, X-ray photoelectron spectroscopy (XPS), and Fourier-transform infrared spectroscopy (FTIR). The effects of factors such as initial solution pH, adsorbent dosage, coexisting ions, and temperature on the adsorption of Pb(II) and Sb(V) were examined. The kinetics, isotherms, thermodynamics, and mechanisms of Pb(II) and Sb(V) adsorption on MnFe@SBA were also investigated.

## 2. Results and Discussion

### 2.1. Characterizations

**Figure 1:**
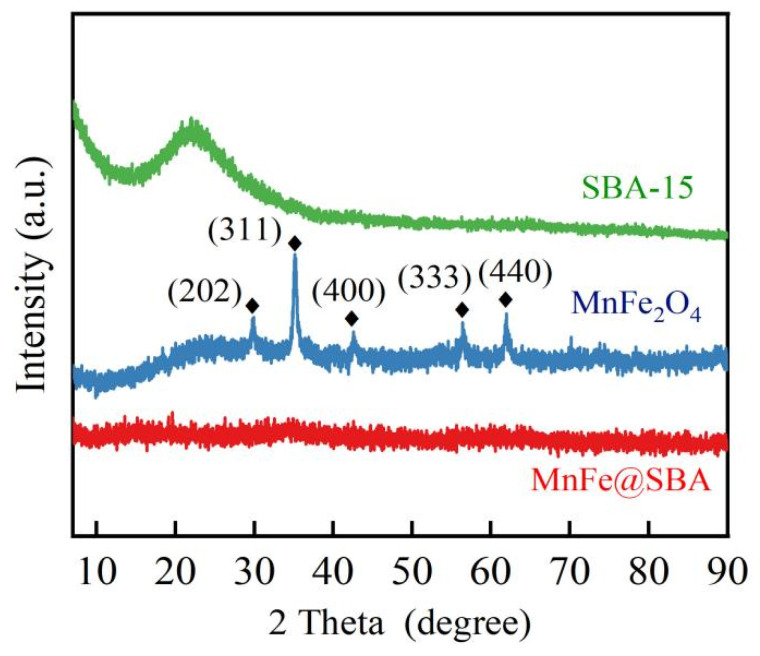
XRD patterns of SBA-15, MnFe_2_O_4_, and MnFe@SBA.

The crystal structures of MnFe_2_O_4_, SBA-15, and MnFe@SBA-0.15 (denoted as MnFe@SBA for short) were determined based on their XRD patterns. As shown in Figure 1, the pure MnFe_2_O_4_ displays five distinct diffraction peaks at 2*θ* values of 29.59°, 34.88°, 42.40°, 56.01°, and 61.51°, corresponding to (202), (311), (400), (333), and (440) planes of standard MnFe_2_O_4_ (PDF#38-0430) [32]. SBA-15 exhibits a broad and faint peak within the 2*θ* range of 20° to 30°, which is consistent with the characteristic peak observed for amorphous silica [33]. Interstingly, no discernible peaks are observed in the XRD patterns of MnFe@SBA, indicating a significantly low degree of crystallization and confirming the amorphous nature of the as-synthesized composite. Moreover, we find that MnFe@SBA exhibits almost no magnetic characteristics, possibly due to the preparation of the material at lower temperatures.

**Figure 2:**
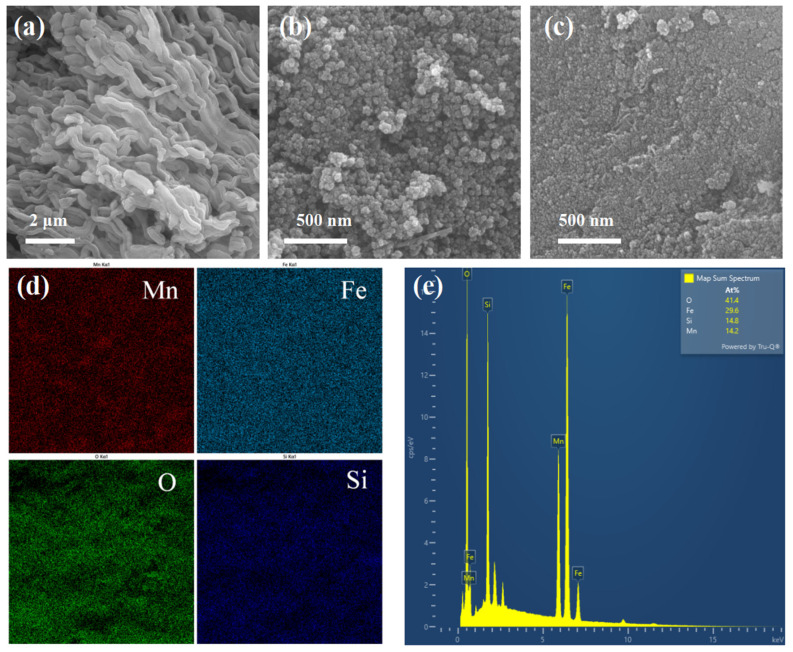
SEM images of (**a**) SBA-15, (**b**) MnFe_2_O_4_, and (**c**) MnFe@SBA; (**d**) EDX elemental mapping images and (**e**) corresponding map sum spectrum of MnFe@SBA.

Figure 2a–c illustrates the surface morphologies of MnFe_2_O_4_, SBA-15, and MnFe@SBA. It is evident that SBA-15 exhibits an ordered channel structure formed by the accumulation of small tubes with lengths ranging from 1 to 2 μm [24]. MnFe_2_O_4_ showcases aggregated spherical nanoparticles measuring approximately 50 nm in size (Figure 2b) [34]. In contrast, the as-synthesized MnFe@SBA displays smaller nanoparticles, with the disappearance of the channel structure of SBA (Figure 2c). From Figure 2d, the elements of Si, O, Mn, and Fe uniformly distribute throughout the whole sample, and the relative mass ratios of O, Fe, Mn, and Si in MnFe@SBA are 41.4%, 29.6%, 14.2%, and 14.8%, respectively (Figure 2e).

**Figure 3:**
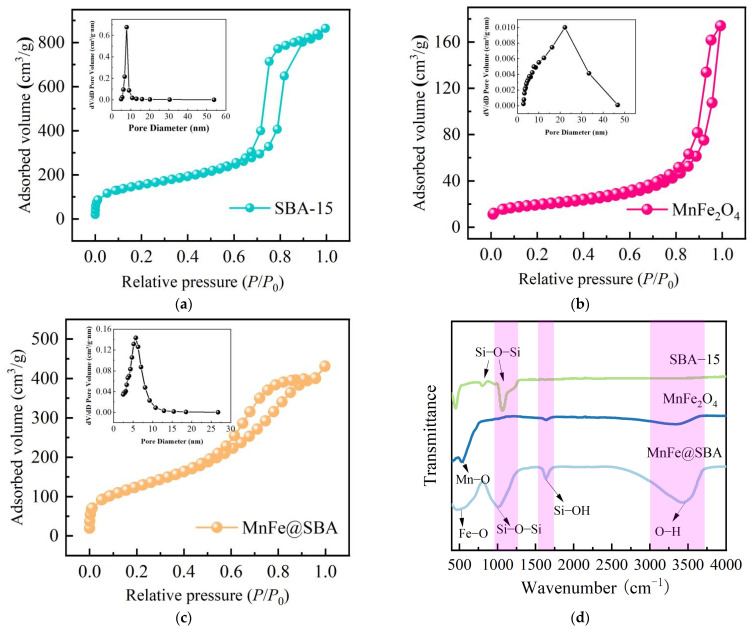
Nitrogen adsorption–desorption isotherms and pore size distributions of (**a**) SBA-15, (**b**) MnFe_2_O_4_, and (**c**) MnFe@SBA; (**d**) FT-IR spectrum of SBA-15, MnFe_2_O_4_, and MnFe@SBA.

To compare the pore structures of SBA-15, MnFe_2_O_4_, and MnFe@SBA, N_2_ adsorption/desorption isotherms were performed, as shown in Figure 3. SBA-15 shows a typical type-IV isotherm with an H1 hysteresis loop at relative pressures ranging from 0.6 to 0.9 (Figure 3a), indicating its classification as a mesoporous material with a pore diameter of 8 nm, as observed from the inset figure [35]. As illustrated in Figure 3b, MnFe_2_O_4_ displays a type-III isotherm accompanied by an H3 hysteresis loop, suggesting enhanced interparticle interactions compared to those between nanoparticles and pollutants. In other words, this observation confirms the propensity of MnFe_2_O_4_ nanoparticles to agglomerate due to the existence of strong interactions. The average pore diameter of MnFe_2_O_4_ ranges from 20 to 30 nm. The as-synthesized MnFe@SBA composite demonstrates a type-IV isotherm with a noticeable H1 hysteresis loop observed between 0.5 and 0.9 (Figure 3c), indicating its mesoporous structure. The pore size distribution curves reveal that the pore diameter of MnFe@SBA falls within the range of 2–10 nm. At the same time, Table S1 further confirms the mesoporous structure of MnFe@SBA. The calculated specific surface area (S_BET_) values follow the order: SBA-15 (537.3 m^2^/g) > MnFe@SBA (405.9 m^2^/g) > MnFe_2_O_4_ (67.9 m^2^/g). Despite some pores in/on SBA-15 being occupied by MnFe_2_O_4_ nanoparticles during the synthesis process, MnFe@SBA maintains a significantly higher specific surface area than MnFe_2_O_4_, facilitating enhanced exposure of adsorption sites for pollutants.

The surface functional groups on MnFe_2_O_4_, SBA-15, and MnFe@SBA were identified via FTIR spectroscopy, as depicted in Figure 3d. A distinctive peak at a wavenumber of 525 cm^−1^ in the FTIR spectrum of MnFe_2_O_4_ is indicative of the presence of Mn–O and Fe–O bonds [36]. In the case of SBA-15, the peaks observed at 1065 and 803 cm^−1^ correspond to characteristic Si–O–Si peaks [33]. The FTIR spectrum of MnFe@SBA demonstrates these aforementioned characteristic peaks, confirming the successful loading of MnFe_2_O_4_ onto SBA-15. Additionally, a broad absorption band at 3424 cm^−1^ appears, corresponding to the O–H stretching vibration. The band at 1634 cm^−1^ can be assigned to Si–OH vibrations.

**Figure 4:**
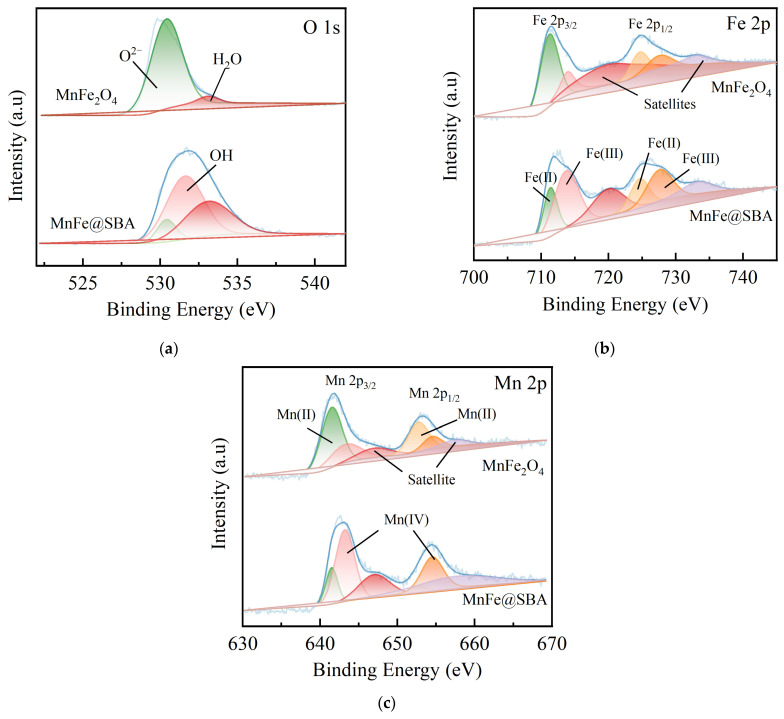
High-resolution XPS spectra: (**a**) O 1s; (**b**) Fe 2p; (**c**) Mn 2p for MnFe_2_O_4_ and MnFe@SBA.

To distinguish the difference before and after SBA-15 loading, the chemical compositions and elemental valence states of MnFe_2_O_4_ and MnFe@SBA were further analyzed using XPS. In Figure 4a, the high-resolution O 1s spectrum of MnFe@SBA can be resolved into three individual peaks at binding energies of 530.4, 531.6, and 533.1 eV, which can be assigned to the lattice oxygen (O^2−^), hydroxyl functional groups (–OH), and adsorbed water (H_2_O) [37], with relative contents of 9.55%, 53.65%, 36.80%, respectively. The incorporation of MnFe_2_O_4_ with SBA-15 results in the decrease in O^2−^ content from 95.17% to 9.55%, along with the enhanced content in –OH to 53.65%. The increased amount of surface –OH could be beneficial for metal ions adsorption. The high-resolution Fe 2p spectrum (Figure 4b) exhibits fitted peaks at 711.3 and 713.9 eV, corresponding to the Fe(II) and Fe(III) orbitals in the form of their respective spin-orbit splitting components, Fe(II)-2p3/2 orbital and Fe(III)-2p3/2 orbital, as well as peaks at 724.6 and 727.6 eV attributed to the Fe(II) and Fe(III) orbitals in terms of their respective spin-orbit splitting components, Fe(II)-2p1/2 orbital and Fe(III)-2p1/2 orbital. Additionally, an oscillating satellite peak is also detectable. The relative proportions of Fe(II) and Fe(III) in MnFe_2_O_4_, as presented in Table S2, are 42.42% and 20.19%, respectively. In the MnFe@SBA sample, the proportion of Fe(III) increased to 47.55%, while the proportion of Fe(II) decreased to 26.04%. The deconvoluted Mn 2p spectrum in Figure 4c shows peaks at 641.5 and 652.7 eV, corresponding to Mn(II), as well as peaks at 643.2 and 654.5 eV, indicating the presence of Mn(Ⅳ) [38,39]. Additionally, a satellite peak is observed at 646.5 eV, which is usually caused by electron transitions related to the metal’s d orbitals. As listed in Table S2, the Mn(II) contents in MnFe_2_O_4_ and MnFe@SBA are determined to be 58.68% and 12.32%, respectively, while approximately 50% of Mn(IV) is detected in MnFe@SBA. These findings confirm the successful loading of MnFe_2_O_4_ onto SBA-15 and the predominant form of Mn(IV) in MnFe@SBA.

### 2.2. Adsorption Performance

#### 2.2.1. Effect of Doping Amount of SBA-15

**Figure 5:**
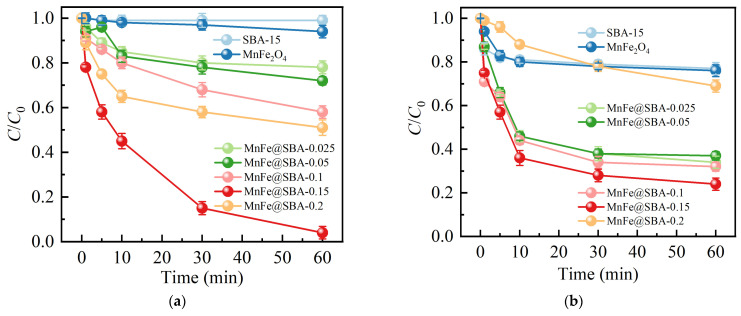
Effect of SBA-15 doping amount on the removal of (**a**) Pb(Ⅱ) and (**b**) Sb(Ⅴ).

The impact of SBA-15 loading amounts on the adsorption performance of MnFe@SBA-*x* for Pb(II) and Sb(V) was investigated. As shown in Figure 5a, both MnFe_2_O_4_ and SBA-15 exhibited negligible removal efficiencies, suggesting their limited adsorption capacity towards Pb(II). The mass loading of SBA-15 significantly influenced the adsorption capacity of MnFe@SBA-*x*. With increasing loading amount from 0.025 to 0.15 g, the removal efficiency of Pb(II) increased from 20% to 100% within 60 min. In Figure 5b, the as-synthesized MnFe@SBA-0.15 achieved the highest adsorption, exceeding 80% for Sb(V) in 120 min. The enhanced adsorption capacity observed upon incorporating SBA-15 can be attributed to the promotion of grain size reduction in the MnFe@SBA composite [40]. However, excessive loading of SBA-15 (0.2 g) resulted in a reduction in removal efficiency to approximately 30% due to the limited adsorption capacity of SBA-15. Therefore, MnFe@SBA-0.15 was selected for subsequent adsorption experiments.

#### 2.2.2. Effect of Solution pH and Coexisting Cations

**Figure 6:**
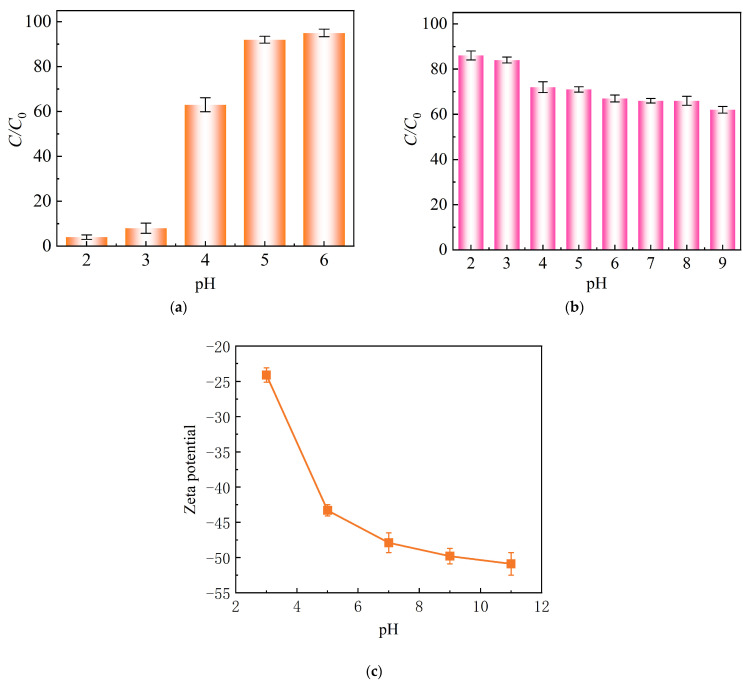
Effect of initial solution pH on removal of (**a**) Pb(Ⅱ) and (**b**) Sb(Ⅴ) by MnFe@SBA. (**c**) Zeta potential of MnFe@SBA at pH ranging from 3 to 11.

The initial solution pH was found to be a crucial factor influencing the effectiveness of adsorption, which can be explained by its effects on both the surface charge of the adsorbent and the speciation of metal ions in the solution [41]. Below pH 6.0, monovalent ionic Pb(II) is predominant in the solution, and small amounts of Pb(OH)^+^ and Pb(OH)_2_ are present at a solution pH of 6–8. Pb(OH)_2_ is the major species when the solution pH ranges from 8 to 11, and Pb(OH)_3_^–^ is present at pH above 8 [42]. In this study, an initial solution pH range of 2–6 was studied to examine how it affected the adsorption of Pb(II) by MnFe@SBA (Figure 6a). The adsorption was found to be negligible within the pH range of 2.0 to 3.0 due to the pronounced acidity. The removal efficiency significantly improved to 63.8% when increasing the solution pH to 4. The zeta potential for MnFe@SBA at different pH values was also recorded in Figure 6c. The surface charge of MnFe@SBA was negative. The poor removal of Pb(II) at extremely acidic conditions was mainly attributed to the competitive effects caused by predominant H^+^ ions competing with Pb(II) [25]. As the solution pH increased from 3 to 11, the potential dropped from −24.2 to −51.2 mV. Thus, the electrostatic attraction force existing between the negatively charged MnFe@SBA and positively charged Pb(II) improved, facilitating the adsorption of Pb(II).

The influence of initial solution pH (2–9) on the removal of Sb(V) was studied, as shown in Figure 6b. Obviously, acidic conditions are more favorable for Sb(V) adsorption. The removal showed a decreasing trend with a gradual increase in solution pH from 3 to 9. This phenomenon may be related to the fact that, at pH higher than 3, Sb(OH)_6_^–^ becomes the predominant species in solution, resulting in an increased electrostatic repulsion force between the negatively charged MnFe@SBA and Sb(OH)_6_^−^, thus inhibiting the adsorption.

Various metal ions and inorganic anions coexist in natural waters. In order to investigate the effectiveness of MnFe@SBA in practical applications, the effects of prevalent metal ions (Na^+^, K^+^, Mg^2+^, and Ca^2+^), as well as divalent heavy metal ions (Zn^2+^, Ni^2+^, and Cd^2+^) on the adsorption of Pb(II) were examined as shown in Figure S2a–g. Additionally, the influences of commonly existing inorganic anions (Cl^−^, NO_3_^−^, CO_3_^2−^, and PO_4_^3−^) on the adsorption of Sb(V) were studied, as shown in Figure S3a–d. It can be observed that Na^+^, K^+^, Mg^2+^, and Ca^2+^ at concentrations of 50 and 100 mg/L had minimal impact on Pb(II) removal. And 100 mg/L Cd^2+^, Ni^2+^, and Zn^2+^ decreased the Pb(II) adsorption from 100% to 80.2%, 82.5%, and 83.2%, respectively [43]. Figure S3a–d demonstrates that high concentrations of Cl^−^, NO_3_^−^, CO_3_^2−^, and PO_4_^3−^ had little effect on the adsorption of Sb(V) by MnFe@SBA. These findings confirm the strong interference resistance of MnFe@SBA in practical application.

#### 2.2.3. Adsorption Kinetics

**Figure 7:**
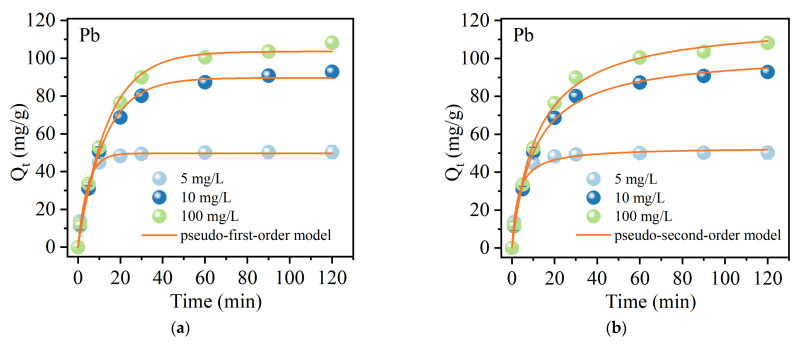

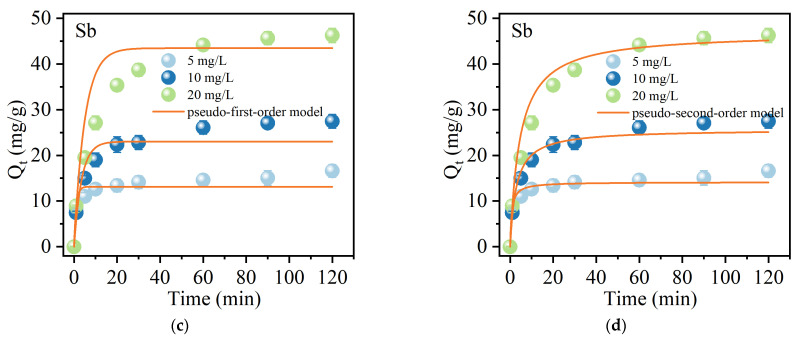
Adsorption kinetics of Pb(II) onto MnFe@SBA fitted by (**a**) pseudo-first-order model and (**b**) pseudo-second-order model; adsorption kinetics of Sb(Ⅴ) onto MnFe@SBA fitted by (**c**) pseudo-first-order model and (**d**) pseudo-second-order model.

The adsorption kinetics of Pb(II) and Sb(V) onto MnFe@SBA-15 were studied at initial concentrations of 5, 10, and 20 mg/L, as shown in Figure 7. A rapid adsorption process occurred within the initial 30 min, with over 80% of the Pb(II) being adsorbed by the adsorbent. Subsequently, the adsorption process gradually approached equilibrium. In the initial stage, abundant vacant sites were available, leading to a high rate of adsorption. As the reaction time exceeded 30 min, the adsorption rate decreased due to a reduced number of available sites for Pb(II) uptake. Once all the sites were occupied, the adsorption reached a dynamic equilibrium. Similarly, MnFe@SBA achieved more than 70% adsorption of Sb(V) within the initial 60 min. The adsorption behavior of Pb(II) and Sb(V) onto MnFe@SBA was modeled using pseudo-first-order and pseudo-second-order kinetic models, represented by the nonlinear Equations (1) and (2) [44]:

(1) $$q_{t}=q_{e}(1-e^{-k_{1}t})$$

(2) $$q_{t}=\frac{k_{2}q_{e}^{2}t}{1+k_{2}q_{e}t}$$

where *q*_e_ and *q*_t_ are the adsorption capacity at equilibrium and at time *t* (min), respectively; *k*_1_ (1/min) is the pseudo-first-order rate constant, and *k*_2_ (g/(mg min)) is the pseudo-second-order rate constant. The nonlinear fitting parameters of the pseudo-first-order and pseudo-second-order kinetic models are summarized in Table 1. Based on the higher coefficients of determination (*R*^2^), the adsorption behavior of Pb(II) and Sb(V) onto MnFe@SBA was better described by the pseudo-second-order kinetic model. Chemisorption was considered as the primary adsorption mechanism. However, the high *R*^2^ values obtained from the pseudo-first-order kinetic model suggested that physical adsorption could not be entirely disregarded, and it might also contribute to the overall adsorption [45].

#### 2.2.4. Adsorption Isotherms

**Figure 8:**
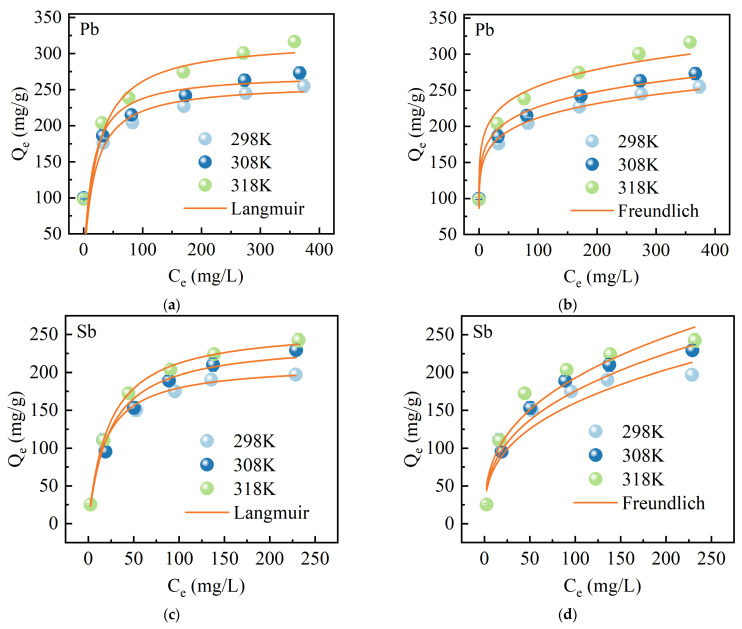
Adsorption isotherms of Pb(II) and Sb(Ⅴ) on MnFe@SBA at temperatures of 298, 308, and 318 K: (**a**,**c**) Langmuir; (**b**,**d**) Freundlich.

To gain a comprehensive understanding of the surface characteristics of the adsorbent, adsorption isotherm studies were conducted at initial concentrations of Pb(II) and Sb(V) ranging from 10 to 400 mg/L, as well as temperatures of 298, 308, and 318 K. The experimental data were fitted using two well-established models: Langmuir and Freundlich isotherms [46]. The Langmuir model assumes a uniform adsorbent surface with no interaction between adjacent sites where ions are adsorbed, thus categorizing the process as monolayer adsorption [47]. On the other hand, the Freundlich model describes the complex nature of multiphase and multilayer adsorption on various types of adsorbents. These models can be represented mathematically as follows [48]:

(3) $$q_{e}=\frac{q_{m}k_{L}C_{e}}{1+k_{L}C_{e}}$$

(4) $$q_{e}=k_{F}C_{e}^{\frac{1}{n}}$$

where *q*_e_ (mg/g) is the equilibrium adsorption capacity, *q*_m_ (mg/g) is the maximum adsorption capacity, *C*_e_ (mg/L) is the equilibrium concentration, *k*_L_ (L/g) is the Langmuir constant associated with adsorption free energy, *k*_F_ ((mg g^−1^)(L mg^−1^)^1/n^) is the Freundlich constant related to adsorption capacity, and *n* is the adsorption energy correlation constant. As depicted in Figure 8, the adsorption of Pb(II) and Sb(V) onto MnFe@SBA exhibited enhanced favorability at elevated temperatures, thereby suggesting an endothermic nature of the adsorption process. According to Table 2, the adsorption of Pb(II) was better fitted by the Freundlich model, as evidenced by higher *R*^2^ values, implying that multilayer adsorption was primarily involved in the adsorption of Pb(II) onto MnFe@SBA. Moreover, a value less than 0.5 for parameter 1/*n* indicated a facile occurrence of adsorption [49]. Conversely, the adsorption behavior of Sb(V) on MnFe@SBA aligned more closely with the Langmuir model, with *R*^2^ values higher than 0.99 at temperatures of 298, 308, and 318 K. The result suggested that monolayered adsorption primarily characterized the interaction between Sb(V) and MnFe@SBA. The maximum adsorption capacities of Sb(V) by MnFe@SBA were found to be 211.49, 241.57, and 260.40 mg/g, respectively.

#### 2.2.5. Adsorption Mechanisms Investigation

**Figure 9:**
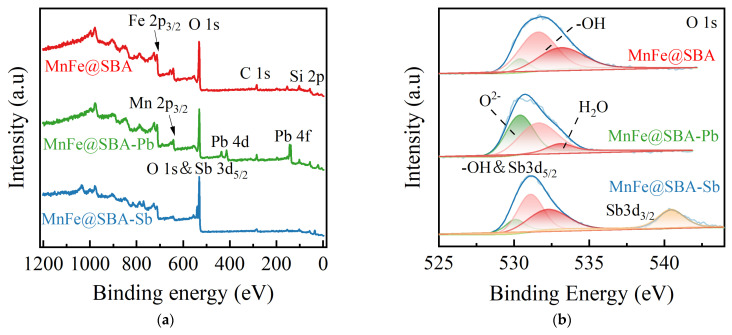

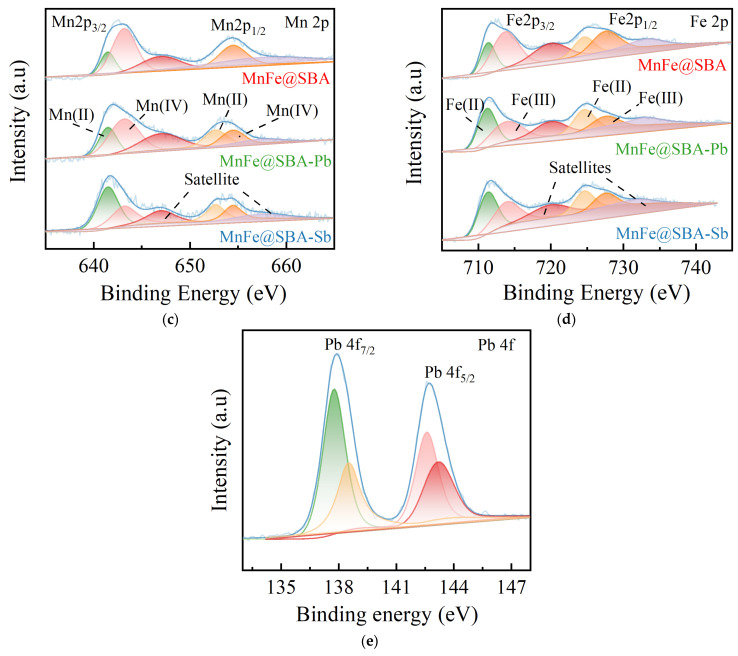
(**a**) XPS survey spectra for the fresh MnFe@SBA, Pb(II) adsorbed MnFe@SBA (MnFe@SBA-Pb), and Sb(V) adsorbed MnFe@SBA (MnFe@SBA-Sb). High-resolution XPS spectra for the fresh MnFe@SBA, MnFe@SBA-Pb, and MnFe@SBA-Sb: (**b**) O 1s, (**c**) Mn 2p and (**d**) Fe 2p. (**e**) High-resolution XPS Pb 4f spectra for MnFe@SBA-Pb.

To gain a deeper understanding of the adsorption mechanisms of Pb(II) and Sb(V) onto MnFe@SBA, XPS analysis was conducted to investigate the surface composition and chemical states of both the fresh and used adsorbent, as illustrated in Figure 9. In the survey spectra (Figure 9a), a distinct peak corresponding to Pb appeared at a binding energy of approximately 140 eV following the adsorption of Pb(II). After the adsorption of Sb(V), there was an evident overlap between O 1s and Sb 3d peaks. These results confirmed the successful adsorption of Pb(II) and Sb(V). The high-resolution O 1s spectrum of the fresh and used MnFe@SBA is presented in Figure 9b. According to Table S3, the adsorption of Pb(II) led to an increase in the proportion of lattice oxygen (O^2−^) from 9.55% to 45.80%, indicating the formation of Pb-O through the interaction between Pb(II) and –OH groups. Consequently, the proportion of –OH dropped from 53.65% to 47.14%, suggesting that –OH on MnFe@SBA played a crucial role in Pb(II) adsorption [50]. Due to the energy overlap between Sb 3d and O 1s, there was a shift in the peak positions of O^2−^, –OH, and H_2_O, leading to an overlap between the –OH peak and Sb 3d_5/2_ [51]. The deconvolution analysis of the Sb 3d_3/2_ peak revealed a single peak at 540.66 eV corresponding to Sb(V), indicating that the adsorption process did not alter the valence state of Sb(V) and was thus straightforward. The relative proportion of Sb 3d_3/2_ was 18.66%, and the proportion of Sb 3d_5/2_ was estimated to be 69.2% of Sb 3d_3/2_ or a calculated value of 12.91%. The initial ratio of –OH to O^2−^ in the fresh adsorbent was 2.35, which subsequently declined to 1.15 after the adsorption of Sb(V), indicating the involvement of –OH.

In the high-resolution Mn 2p XPS spectrum (Figure 9c), the relative proportion of Mn(II) peak at 641.5 and 652.7 eV increased from 12.43% to 28.89% upon Pb(II) adsorption. Concurrently, the proportion of the Mn(IV) peak at 643.2 and 654.5 eV decreased from 50% to 44.24%. This phenomenon was likely due to the release of Mn(IV) from the interlayer, which subsequently re-adsorbed onto the surface as Mn(II) [52]. Analogous alterations were observed in the XPS spectrum of the Sb(V) adsorbed MnFe@SBA, suggesting that Mn(IV) was similarly released from the interlayer and adsorbed on the surface as Mn(II). This process enhanced the zeta potential and facilitated the formation of a Mn–Sb–O complex via coordination with Sb(V) [53].

As illustrated in Figure 9d, following the adsorption of Pb(II) and Sb(V), the relative proportion of Fe(II) in the adsorbent increased while the proportion of Fe(III) decreased. The result strongly suggested the occurrence of electron transfer and redox reactions throughout the process. Figure 9e represents the high-resolution XPS spectrum of Pb 4f, which can be deconvoluted into two individual peaks at 137.77 and 142.60 eV, corresponding to Pb 4f_7/2_ and Pb 4f_5/2_, respectively. Further, the peaks at 138.50 and 143.20 eV are indicative of Pb=O and Pb−O species [54]. These findings can be attributed to the complex interaction between MnFe@SBA and Pb(II).

**Figure 10:**
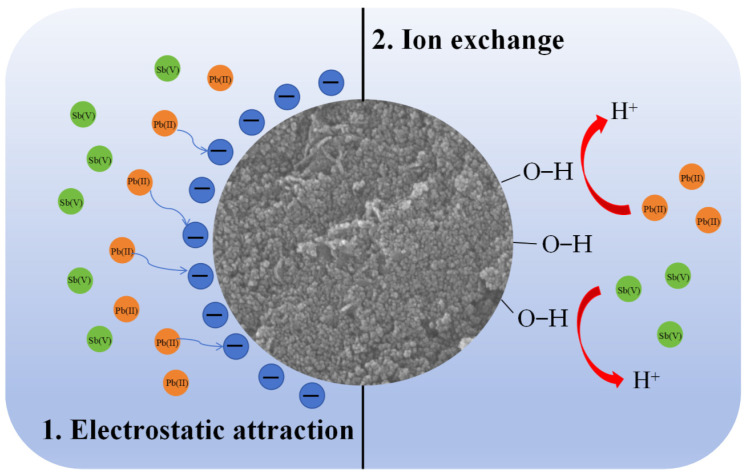
Diagram of the potential mechanisms of Sb(V) and Pb(II) adsorption by MnFe@SBA.

In summary, the adsorption mechanisms of Pb(II) and Sb(V) onto MnFe@SBA were found to exhibit similarities and consist of two steps, as illustrated in Figure 10. Initially, an electrostatic attraction occurred between MnFe@SBA and Pb(II)/Sb(V), which played a significant role in contributing to the rapid adsorption process. Subsequently, –OH groups on MnFe@SBA formed the inner-sphere complexes with Pb(II). In the case of Sb(V) adsorption, MnFe@SBA enhanced the surface zeta potential via the interlayer release and re-adsorption of Mn(IV), thereby attracting Sb(V) and forming Sb–O–Mn complexes.

## 3. Chemicals and Methods

### 3.1. Chemicals

SBA-15 was purchased from Jiangsu Xianfeng Nanomaterial Technology Co., Ltd. (Nanjing, China). Tetrahydrate manganese chloride (MnCl_2_·4H_2_O), sodium hydroxide (NaOH), and hexahydrate iron chloride (FeCl_3_·6H_2_O) were obtained from Shanghai Guoyao Chemical Reagent Co., Ltd. (Shanghai, China). All chemicals were of analytical purity and were used as received without further purification. All solutions were prepared using ultrapure water produced using an ultrapure water purification system (Veolia Water Solutions and Technologies, Singapore).

### 3.2. Synthesis of MnFe@SBA

MnFe@SBA was synthesized using a simple hydrothermal method, as shown in Figure S2. First, 2 mmol of MnCl_2_·4H_2_O and 4 mmol of FeCl_3_·6H_2_O were dissolved in a beaker to form 100 mL of mixed solution. Subsequently, 10 mL of NaOH solution was added, and the resulting solution was stirred continuously for 10 min. SBA-15 with mass loads from 0.025 to 0.2 g was then added. After being agitated for 10 min, the solution was placed into a Teflon-lined stainless-steel autoclave and heated to and held at a temperature of 100 °C for 24 h. The autoclave was then cooled naturally to room temperature. The precipitate was collected, washed with deionized water, and dried in an oven at 60 °C for 12 h. The product is referred to hereinafter as MnFe@SBA-*x*, where *x* denotes the loading amount of SBA-15, and the loading capacity of MnFe_2_O_4_ is maintained at 0.46 g. The pure MnFe_2_O_4_ was synthesized as well without the addition of SBA-15.

### 3.3. Characterizations

The surface morphologies of the samples were observed using a scanning electron microscope (MIRA3 LMH, TESCAN Group a.s., Brno, Czech). Elemental analyses of the adsorbents were performed using energy-dispersive X-ray spectrometry (EDS, One Max 20, UK). X-ray diffraction (XRD) analysis was performed using an X-ray diffractometer (Rigaku SmartLab SE, Akishima, Japan). Fourier-transform infrared spectroscopy (FTIR) was performed using an analytical FTIR spectrometer (Thermo Fisher Scientific Nicolet iS50, Waltham, MA, USA). The BET surface areas and pore size distribution of the N_2_ adsorption–desorption isotherms were measured using an analytical instrument (Micromeritics ASAP 2460, Norcross, GA, USA). X-ray photoelectron spectroscopy (XPS) spectra were characterized by an XPS spectrometer (AXIS SUPRA+, Shimadzu, Kyoto, Japan). The pH values of the solutions were measured using a pHs-3C digital pH meter (Shanghai Precision Scientific Instruments Co., Ltd., Shanghai, China). The zeta potentials of the adsorbents were determined using a Zetasizer Nano ZS90 (Malvern Instruments, Malvern, UK).

### 3.4. Batch Adsorption Experiments

Typically, at room temperature, 10 and 30 mg of MnFe@SBA were dispersed in 100 mL of Pb(II) (10 mg/L) and Sb(V) solutions (10 mg/L), respectively. At preset time intervals spanning 0 to 120 min, 0.5 mL of water sample was extracted and filtered using a syringe filter membrane (0.22 µm) immediately. The concentration of residue Pb(II) and Sb(V) ions in the solution was determined using atomic absorption spectrophotometry (AA-6300; Shimadzu, Kyoto, Japan). The removal efficiency and adsorption capacity were computed using the equations of $Q_{e}=\frac{(C_{0}-C_{e})\times V}{m}$ and $R=\frac{C_{0}-C_{e}}{C_{0}}\times 100\%$, respectively [55], where *C*_0_ and *C*_e_ (mg/L) are the initial and equilibrium concentrations in the solution, respectively; *V* (L) is the solution volume; *m* (g) is the adsorbent mass; *Q*_e_ (mg/g) is the adsorption capacity at equilibrium; and *R* (%) is the removal efficiency. To examine the impact of pH on the adsorption, a solution containing 10 mg/L Pb(II) or Sb(V) was adjusted to the desired pH range using 1 M HCl and NaOH. The interference of commonly coexisting cations (Na^+^, K^+^, Ca^2+^, Mg^2+^, Cd^2+^, Ni^2+^ and Zn^2+^) and anions (Cl^−^, NO_3_^−^, CO_3_^2−^, and PO_4_^3−^) on the adsorption was also investigated.

To study the adsorption kinetics, 10 and 30 mg of MnFe@SBA were added to Pb(II) and Sb(V) solutions with initial concentrations of 5, 10, and 20 mg/L at a constant pH of 6, respectively. To study the adsorption isotherms, 10 and 30 mg of MnFe@SBA were added to Pb(II) and Sb(V) solutions with initial concentrations ranging from 10 to 400 mg/L at a pH of 6, respectively. The adsorption of Pb(II) and Sb(V) using MnFe@SBA at temperatures of 25, 35, and 45 °C were studied to understand the adsorption thermodynamics.

## 4. Conclusions

In this work, a novel amorphous MnFe@SBA composite was synthesized through the in situ growth of MnFe_2_O_4_ on SBA-15 to effectively remove heavy metals (Pb(II) and Sb(V)) from aqueous solutions. Both MnFe_2_O_4_ and SBA-15 demonstrated limited adsorption capacities for Pb(II) and Sb(V). However, by optimizing the material synthesis and adsorption conditions, the maximum adsorption capacities of MnFe@SBA for Pb(II) and Sb(V) at 318 K were significantly enhanced to 329.86 mg/g and 260.40 mg/g, respectively. The adsorption process for both Pb(II) and Sb(V) by MnFe@SBA was found to be endothermic and spontaneous. The adsorption of Pb(II) onto MnFe@SBA was well-fitted by the pseudo-second-order kinetic and Freundlich models, while the adsorption of Sb(V) was well-described by the pseudo-second-order kinetic and Langmuir models. Mechanistic studies indicated that the adsorption of Pb(II) and Sb(V) onto MnFe@SBA involved two primary steps: electrostatic attraction and complexation. Initially, rapid adsorption occurred due to the electrostatic attraction between MnFe@SBA and metal ions. Subsequently, –OH groups on MnFe@SBA formed inner-sphere complexes with Pb(II). For Sb(V) adsorption, MnFe@SBA enhanced its surface zeta potential through the interlayer release and re-adsorption of Mn(IV), facilitating the formation of Sb–O–Mn complexes.

## Figures and Tables

**Table 1:** Parameters of pseudo-first-order and pseudo-second-order kinetic models for the adsorption of Pb(II) and Sb(Ⅴ) onto MnFe@SBA.

	C0 (mg/L)	Pseudo-First-Order Model			Pseudo-Second-Order Model		
		K1 (L/min)	Qe (mg/g)	R2	K2 (g/(mg min))	Qe (mg/g)	R2
Pb(II)	5	0.22611	49.68	0.9983	0.00663	53.07	0.9983
	10	0.08088	89.61	0.9983	0.00092	103.27	0.9986
	20	0.07198	103.58	0.9982	0.00069	120.08	0.9988
Sb(Ⅴ)	5	1.00037	13.15	0.9928	0.09955	14.15	0.9969
	10	0.28702	23.02	0.9680	0.01387	25.69	0.9920
	20	0.20328	43.47	0.9765	0.00474	46.84	0.9937

**Table 2:** Parameters of Langmuir and Freundlich isotherms models for the adsorption of Pb(II) and Sb(Ⅴ) onto MnFe@SBA.

	T (K)	Langmuir			Freundlich		
		K_L_ (L/min)	Q_m_ (mg/g)	R^2^	1/*n*	K_F_ [(mg/g)(L/mg)1/n]	R^2^
Pb(II)	298	0.0569	258.77	0.852	0.130	116.41	0.977
	308	0.0691	271.85	0.885	0.129	125.19	0.991
	318	0.0447	329.86	0.872	0.126	143.09	0.974
Sb(Ⅴ)	298	0.0562	211.49	0.998	0.349	31.95	0.863
	308	0.0434	241.57	0.993	0.357	33.87	0.856
	318	0.0438	260.40	0.999	0.356	37.45	0.823

## Data Availability

Data is contained within the article and Supplementary Materials.

## Supplementary Materials

The following supporting information can be downloaded at: https://www.mdpi.com/article/10.3390/molecules30030679/s1, Figure S1: Synthesis procedure for amorphous MnFe@SBA; Figure S2: Effects of (a) Na^+^, (b) K^+^, (c) Ca^2+^, (d) Mg^2+^, (e) Cd^2+^, (f) Ni^2+^, (g) Zn^2+^ on the removal of Pb(Ⅱ) by MnFe@SBA; Figure S3: Effects of (a) Cl^−^, (b) NO_3_^−^, (c) CO_3_^2−^, (d) PO_4_^3−^ on the removal of Sb(Ⅴ) by MnFe@SBA; Table S1: Textural properties of the SBA-15, MnFe_2_O_4_, and MnFe@SBA composites; Table S2: Peaks information of O 1s, Fe 2p, and Mn 2p; Table S3: Peaks information of O 1s, Fe 2p, and Mn 2p.
