# Supplementary material for: Synthesis of Amorphous MnFe@SBA Composites for Efficient Adsorptive Removal of Pb(Ⅱ) and Sb(V) from Aqueous Solution
Source: Molecules. 2025 Feb 4;30(3):679. doi: 10.3390/molecules30030679 (PMC11820195; doi:10.3390/molecules30030679)
Supplement: Supplementary file 1 [file molecules-30-00679-s001.zip › molecules-3406297-supplementary.pdf]

Supporting materials

for

Synthesis of Amorphous MnFe@SBA Composites for Efficient Adsorptive Removal of Pb(II) and Sb(V) from Aqueous Solution

Zhou Shi, Aogui Zhu, Fan Chen, Yishu Cai *, Lin Deng *

Hunan Engineering Research Center of Water Security Technology and Application, College of Civil Engineering, Hunan University, Changsha 410082, China; shiz61@hnu.edu.cn (Z.S.); zhuaogui1@hnu.edu.cn (A.Z.); chenfan@cmie.cn (F.C.)

* Correspondence: yishu.cai@hnu.edu.cn (Y.C.); lindeng@hnu.edu.cn (L.D.)

This file contains 8 pages (including the cover page), 3 figures, and 3 tables.

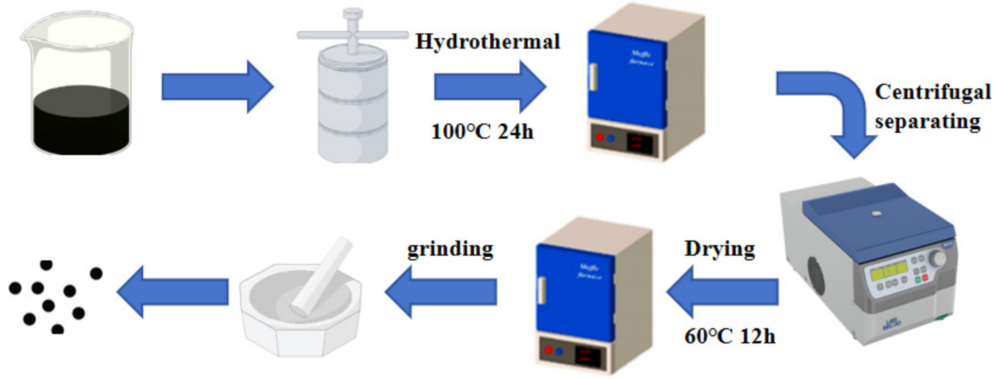

Figure S1. Synthesis procedure for amorphous MnFe@SBA.

Table S1. Textural properties of the SBA-15, $MnFe_2O_4$, and MnFe@SBA composites.

Sample	S_{BET} (m^2/g)	$S_{external}$ (m^2/g)	$V_{mesopore}$ (cm^3/g)	$V_{micropore}$ (cm^3/g)
SBA-15	537.2565	491.8858	1.337462	0.018234
$MnFe_2O_4$	67.9062	56.163	0.269258	0.004698
MnFe@SBA	405.9077	352.1925	0.665244	0.079617

Table S2. Peaks information of O 1s, Fe 2p, and Mn 2p.

		Binding energy (eV)	Relative proportions (%)	
			$MnFe_2O_4$	MnFe@SBA
O	O^{2-}	530.4	95.17	9.55
	-OH	531.6	-	53.65
	H_2O	533.1	4.83	36.80
Fe	Fe(II)	711.3	32.15	14.40
	Fe(III)	713.9	11.71	30.34
	Fe(II)	724.6	10.27	11.64
	Fe(III)	727.6	8.48	17.21
Mn	Mn(II)	641.5	39.44	9.68
	Mn(IV)	643.2	18.41	31.33
	Mn(II)	652.7	19.24	2.64
	Mn(IV)	654.5	7.46	18.67

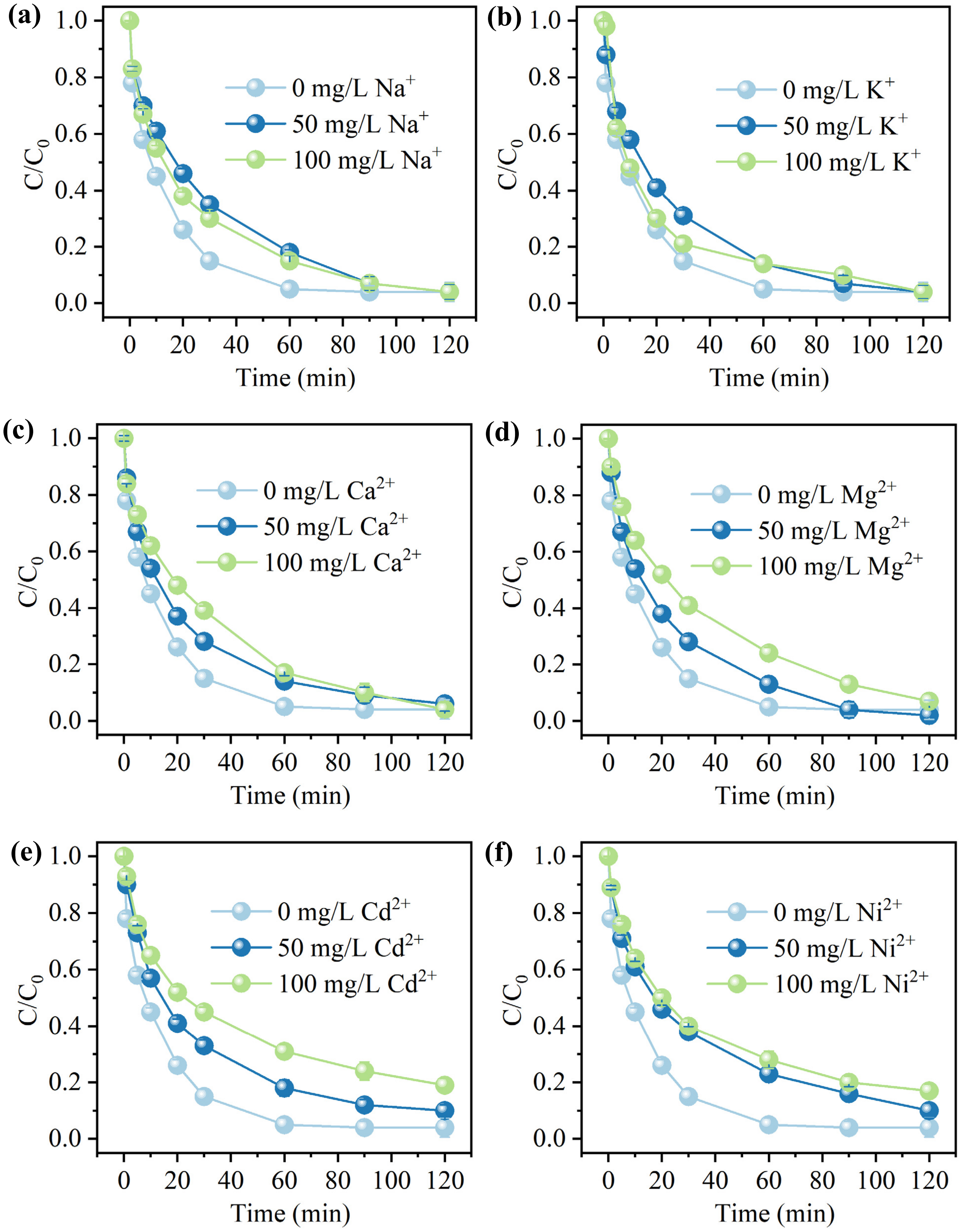
(a)
0 mg/L Na+
50 mg/L Na+
100 mg/L Na+
(b)
0 mg/L K+
50 mg/L K+
100 mg/L K+
(c)
0 mg/L Ca2+
50 mg/L Ca2+
100 mg/L Ca2+
(d)
0 mg/L Mg2+
50 mg/L Mg2+
100 mg/L Mg2+
(e)
0 mg/L Cd2+
50 mg/L Cd2+
100 mg/L Cd2+
(f)
0 mg/L Ni2+
50 mg/L Ni2+
100 mg/L Ni2+
C/C0
Time (min)
0.0
0.2
0.4
0.6
0.8
1.0
0
20
40
60
80
100
120

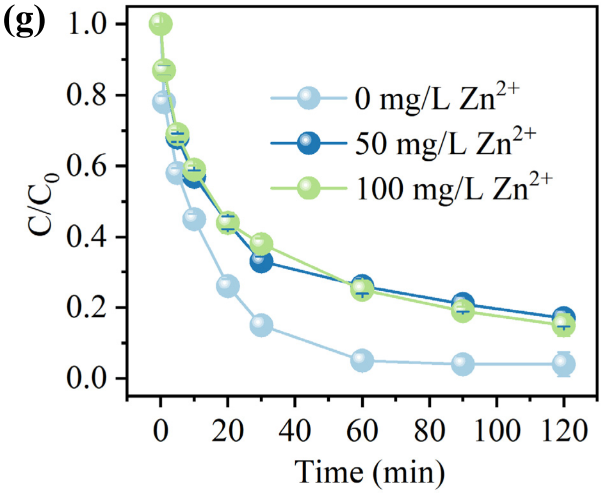

Figure S2. Effects of (a) Na^+ , (b) K^+, (c) Ca^{2+}, (d) Mg^{2+}, (e) Cd^{2+}, (f) Ni^{2+}, (g) Zn^{2+} on the removal of Pb(II) by MnFe@SBA.

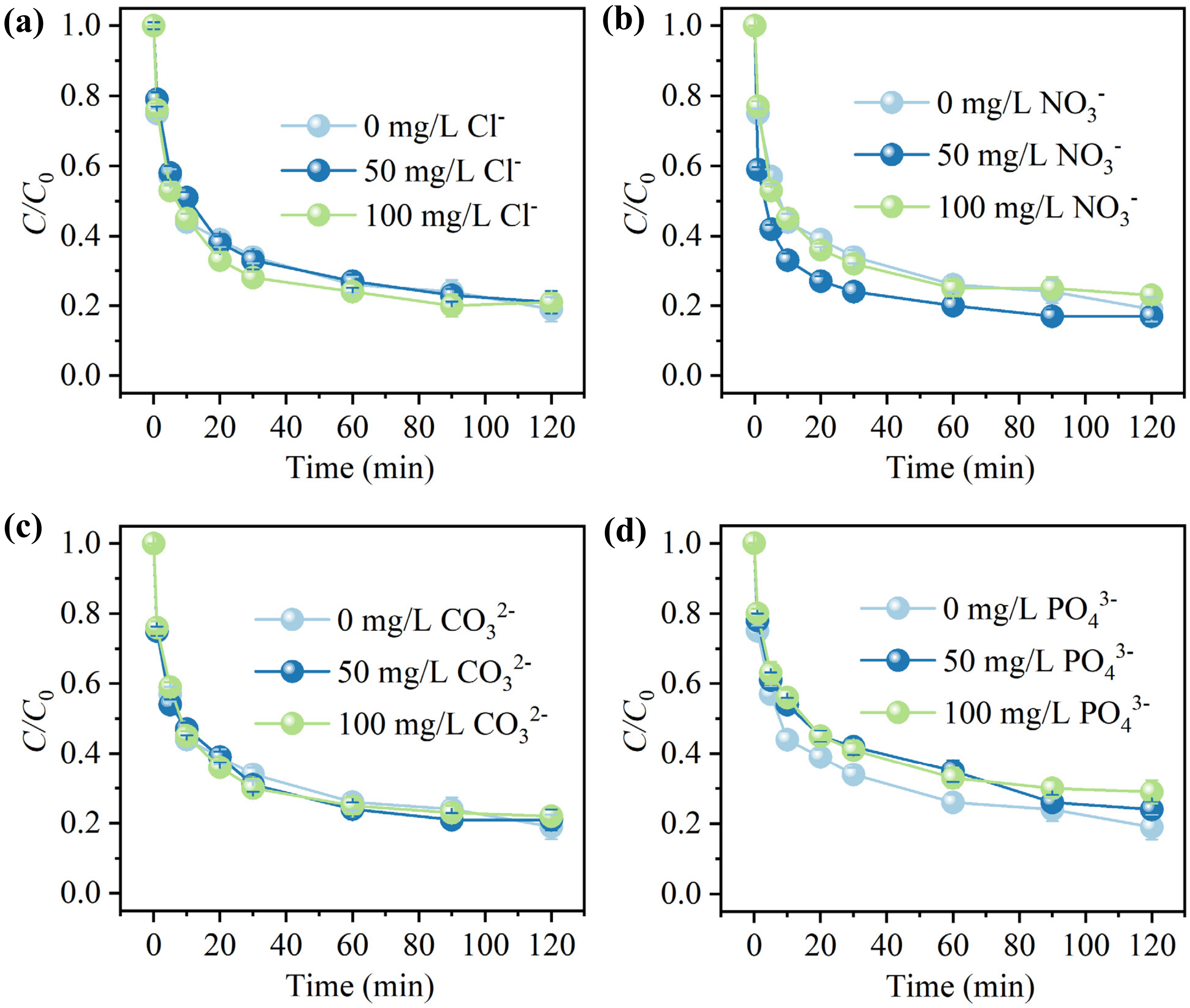

Figure S3. Effects of (a) Cl^-, (b) NO_3^-, (c) CO_3^{2-}, (d) PO_4^{3-} on the removal of Sb(V) by MnFe@SBA.

Table S3. Peaks information of O 1s, Fe 2p, and Mn 2p.

		Binding energy (eV)	Relative proportions (%)		
			MnFe@SBA	MnFe@SBA-Pb(II)	MnFe@SBA-Sb(V)
O	O^{2-}	530.4	9.55	45.80	-
	-OH	531.6	53.65	47.14	-
	H_2O	533.1	36.80	7.06	-
Fe	Fe(II)	711.3	14.40	23.25	27.01
	Fe(III)	713.9	30.34	21.59	25.84
	Fe(II)	724.6	11.64	15.23	14.74
	Fe(III)	727.6	17.21	10.50	10.49
Mn	Mn(II)	641.5	9.68	15.97	31.40
	Mn(IV)	643.2	31.33	33.24	21.61
	Mn(II)	652.7	2.64	12.92	13.21
	Mn(IV)	654.5	18.67	11.00	10.82
